# Transcriptomic, Proteomic, and Metabolic Profiles of *Catalpa bungei* Tension Wood Reveal New Insight Into Lignin Biosynthesis Involving Transcription Factor Regulation

**DOI:** 10.3389/fpls.2021.704262

**Published:** 2021-11-15

**Authors:** Yao Xiao, Juanjuan Ling, Fei Yi, Wenjun Ma, Nan Lu, Tianqing Zhu, Junhui Wang, Kun Zhao, Huiling Yun

**Affiliations:** ^1^State Key Laboratory of Tree Genetics and Breeding, Key Laboratory of Tree Breeding and Cultivation of National Forestry and Grassland Administration, National Innovation Alliance of Catalpa bungei, Research Institute of Forestry, Chinese Academy of Forestry, Beijing, China; ^2^Luoyang Academy of Agriculture and Forestry Sciences, Luoyang, China; ^3^Xiaolongshan Research Institute of Forest Science and Technology, Tianshui, China

**Keywords:** tension wood, *Catalpa bungei*, lignin metabolism, transcription factor, regulatory network

## Abstract

Lignin is a complex polymer in plant cell walls whose proportion is second only to that of cellulose and plays an important role in the mechanical properties of wood and stress resistance of plants. Here, we induced tension wood (TW) formation in *Catalpa bungei* by artificial bending and analyzed the lignin metabolism of the TW. LC-MS analysis showed that a significantly higher content of coniferyl aldehyde was observed in the TW cell wall than in the opposite wood (OW) and normal wood (NW) cell walls. TW had significantly lower contents of coniferyl alcohol than OW and NW. Raman spectroscopy results indicated that TW had lower total lignin than OW and NW. The transcription and translation levels of most of the differentially expressed genes (DEGs) involved in lignin monomer biosynthesis indicated upregulation in TW/OW and TW/NW. We found no significant difference in the transcription levels of three collision gases (CADs) between TW and OW or between NW, but their translation levels were significantly downregulated in TW, suggesting post-transcriptional control for *CAD*. We predicted and analyzed transcription factors that could target DEGs involved in lignin monomer biosynthesis in TW. Based on the analysis of the relationships of targeting and coexpression, we found that NAC (evm.model.group1.695) could potentially target *4CLs* and *CCoAOMT*, that HD-Zip (evm.model.group7.1157) had potential targeting relationships with *CCoAOMT*, *F5H*, and *CCR*, and that their expression levels were significantly positive. It is speculated that the upregulation of NAC and HD-ZIP transcription factors activates the expression of downstream target genes, which leads to a significant increase in coniferyl aldehyde in TW. However, the decrease in total lignin in TW may be caused by the significant downregulation of CAD translation and the significant decrease in precursors (coniferyl alcohol). Whether the expression of CAD genes is regulated by post-transcriptional control and affects TW lignin metabolism needs further study.

## Introduction

Wood, the secondary xylem of trees, is a multipurpose renewable material and is commonly used in papermaking, architecture, furniture manufacturing, and energy production due to its varied properties (Nakahama et al., 2018; Papadopoulos et al., 2019). Wood formation is considered secondary growth and involves the formation of vascular tissue, secondary cell wall (SCW), lignification, programmed cell death, and heartwood formation (Tian et al., 2007). Wood quality is mainly determined by cellulose, hemicellulose, and lignin contents (Nakahama et al., 2018). Lignification can increase the hardness and mechanical supporting force of the secondary xylem cell wall and enhance the ability of plants to transport water over long distances and resist biological and abiotic stresses (Vance et al., 1980; Zhao, 2016; Hu et al., 2019). Lignin is related to the properties of pulp and paper (Liang et al., 2020). Therefore, it is of great significance to study the distribution, synthesis and regulation of lignin in xylem for the genetic improvement of wood properties.

The lignin synthesis pathway has been clearly elucidated (Zhao, 2016). Generally, lignin in higher plants is formed by the oxidative polymerization of three kinds of lignin monomers (p-hydroxyphenyl lignin, guaiacyl lignin, and syringyl lignin). Studies have found that lignin monomer synthesis pathways in different plants are significantly different (Li et al., 2014). For example, *caffeoyl shikimate esterase* (*CSE*) is a key gene for lignin monomer synthesis in *Arabidopsis thaliana*, and mutation of this gene will greatly reduce the lignin content in *A. thaliana* (Shen et al., 2013; Vanholme et al., 2013). Overexpression of the *F5H* gene in *A. thaliana* with a defective *COMT* gene leads to enrichment in unusual lignin (5H lignin) (Weng et al., 2010). These results indicate the flexibility of the lignification process. This flexibility is the starting point for the artificial regulation of lignin biosynthesis and will increase the possible applications of many economically important plants. Research has found that reducing the expression of the *4CL* gene during lignin synthesis in poplar will reduce Klason lignin by 30%, which reduces the elasticity modulus by 40% (Horvath et al., 2010). However, increasing *4CL* gene expression in poplar will increase lignin content by approximately 40%, reduce other carbohydrate contents and significantly increase wood strength (Hu et al., 2019). Altering the expression of genes in the lignin synthesis pathway has been shown to significantly influence the chemical composition of cell walls. Wu et al. (2019) confirmed that lignin monomer biosynthesis was strictly regulated by genes in synergy. They found that downregulation of *F5H* in COMT-RNAi transgenic switchgrass inhibits S lignin biosynthesis and increases guaiacyl lignin units. In contrast, overexpression of *F5H* in COMT-RNAi transgenic plants will reduce the number of G units. Deficient lignin biosynthesis could be partially compensated for or completely recovered with different degrees of *COMT* downregulation. These factors make it possible to artificially and precisely regulate the synthesis of lignin monomers.

Wood formation is a complicated process under the control of a large set of transcription factors (Sun et al., 2021). Lignin biosynthesis and lignification are two of the steps in wood formation. Transcriptional regulation is important for lignin synthesis. Studies have indicated that the lignin metabolic pathway usually has the same transcriptional regulation mode as cellulose and hemicellulose biosynthesis (Zhao and Dixon, 2011; Nakano et al., 2015). NAC transcription factors are usually the first-layer managers in the regulation network of wood chemical composition biosynthesis. NACs can target a number of *MYB* genes (second-layer managers) and promote their direct or indirect regulation of the formation of secondary walls for plants, including lignin biosynthesis. In *A. thaliana*, *MYB46*, *MYB58*, and *MYB63* are targeted by SND1, NST1, and NST2 (Zhou et al., 2009; Zhong et al., 2010). In *atnst1-1* and *atnst3-1-1* double mutants, secondary wall thickening was completely inhibited, and some lignin synthesis pathway genes were also inhibited (Mitsuda et al., 2005). Moreover, lignin biosynthesis and cellulose deposition genes were highly expressed in the *myb75-1* mutant with thickening of the fiber cell wall, increasing the lignin content and changing the S/G ratio in the stem (Bhargava et al., 2010). The *PtrWND* genes could induce the expression of wood biosynthetic genes, including related transcription factors and structural genes, leading to ectopic deposition of lignin in poplars (Zhong et al., 2010). Furthermore, several studies have shown that PtrMYB3 and PtrMYB20 can activate the lignin biosynthesis pathway (McCarthy et al., 2010). In addition to the NAC and MYB transcription factors, an increasing number of transcription factors have been found to be involved in wood formation. For example, homeobox genes are associated with lignification in bamboo (Xu et al., 2019), and *PtrHB7* expression enhances the differentiation of cambial cells toward xylem cells in Populus (Zhu et al., 2013). HD-ZIP also regulates lignin biosynthesis in plants (Sun et al., 2020). These studies suggest a new level of complexity in lignin biosynthesis regulation.

*Catalpa bungei* is native to China and is an economically important ring-porous tree species with high-quality wood (Li et al., 2019). Traditional breeding for rapid growth of *C. bungei* has been carried out for years (Xiao et al., 2019). However, currently, the forest industry demands not only high yields but also high quality. Thus, it is necessary to study the mechanism of wood formation in *C. bungei*. Recently, with the development of high-throughput sequencing techniques, multiomics analysis has played an important role in the selection of functional genes and the construction of gene expression regulatory networks for wood formation (Shinya et al., 2016; Bygdell et al., 2017; Wang et al., 2018). We combined transcriptome, proteome, and confocal Raman imaging techniques to analyze the xylem of tension wood (TW), opposite wood (OW), and normal wood (NW) in *C. bungei* and identified the key genes that regulate lignin biosynthesis, providing new insight for wood property improvement by genetic engineering in *C. bungei*.

## Materials and Methods

### Plant Material

One-year-old *C. bungei* clone 8,402 was planted at the experimental base in Luoyang city, Henan Province. We used artificial bending to induce TW formation in July. The method was as follows: one end of twine was tied to the top of the plant, and the other end was tied to a heavy object. The object was then dragged to bend the plant. The plants were kept in a bent state with a consistent angle of 45° in the same direction. Refer to the literature for a diagram of the bending treatment mode (Xiao et al., 2020). After 3 months of treatment, wood samples were collected at a height of 60 cm, and xylem was obtained to make tissue sections. The sections were double stained with saffron-fast green, and the detailed staining steps are described in the literature (Xiao et al., 2020). The staining results were used to identify the TW and OW.

### Ultrastructure Observation

Sample preparation was carried out essentially as previously described by Xiao et al. (2020). The xylem samples were cut using a razor blade and immediately fixed in 3% (w/v) glutaraldehyde. Samples were dehydrated in an ethanol series (30, 50, 70, and 100%) and embedded in Spurr resin at 60°C. Transverse resin sections of xylem were cut using a diamond knife for ultrastructural observation under a transmission electron microscope (HITACHI HT-7700, Tokyo, Japan).

### Confocal Raman Mapping

A LabRam Xplora confocal Raman microscope (Horiba Jobin–Yvon, Paris, France) with an Olympus confocal microscope (Olympus, Tokyo, Japan) and a linearly polarized 532 nm laser (Ventus VIS 532, Laser Quantum, Chester, United Kingdom) was used to measure the sample size. The power was set to 8 mW. Point-by-point scanning microprobe imaging was employed to acquire spectral data. The parameter settings were as follows: 1,200 mm^–1^ grating, 100 μm slit width, 300 μm numerical aperture, 0.7 μm scanning step, 1 s single-point spectral acquisition time, and 2 cm^–1^ spectral resolution. Every 15 spectra were acquired and averaged. Data were acquired and analyzed by LabSpec6 software. The entire experiment was carried out at a constant 25°C.

### Liquid Chromatography-Mass Spectrometry (LC-MS) Determination of Metabolites in the Lignin Monomer Biosynthetic Pathway

A total of 100 mg of sample powder was weighed and extracted overnight at 4°C with 1.0 mL 70% aqueous methanol. Following centrifugation at 10,000 × *g* for 10 min, the extracts were subjected to SPE (CNWBOND Carbon-GCB solid phase extract (SPE) cartridge, 250 mg, 3 mL; ANPEL, Shanghai, China), and the eluates were filtered (SCAA-104, 0.22 μm pore size; ANPEL) before LC-MS analysis.

The sample extracts were analyzed using an LC-electrospray ionization (ESI)-MS/MS system [high performance liquid chromatography (HPLC), Shim-pack UFLC Shimadzu CBM30A system, Kyoto, Japan; MS, Applied Biosystems, Carlsbad, CA, United States]. The analytical conditions for HPLC were as follows: column, Waters ACQUITY UPLC HSS T3 C18 column (1.8 μm, 2.1 mm × 100 mm); solvent system, water (0.04% acetic acid): acetonitrile (0.04% acetic acid); gradient program, 100:0 V/V at 0 min, 5:95 V/V at 11.0 min, 5:95 V/V at 12.0 min, 95:5 V/V at 12.1 min, and 95:5 V/V at 15.0 min; flow rate, 0.40 mL/min; temperature, 40°C; and injection volume, 2 μL. The effluent was alternatively connected to an electrospray ionization (ESI)-triple quadrupole-linear ion trap (Q TRAP)-MS.

Linear ion trap (LIT) and triple quadrupole (QQQ) scans were acquired on a QQQ-LIT mass spectrometer (Q TRAP), API 6500 Q TRAP LC-MS/MS system, equipped with an ESI Turbo Ion-Spray interface, operating in positive ion mode and controlled by Analyst 1.6 software (AB Sciex). The ESI source operation parameters were as follows: ion source, turbo spray; source temperature, 500°C; ion-spray voltage (IS), 5,500 V; ion source gas I (GSI), 55 psi; gas II (GSII), 60 psi; curtain gas (CUR), 25.0 psi; and collision gas (CAD), high. Instrument tuning and mass calibration were performed with 10 and 100 μmol/l polypropylene glycol solutions in the 6500 Q TRAP and LIT modes, respectively. Q TRAP scans were acquired as multiple reaction monitoring (MRM) experiments with the CAD (nitrogen) set to 5 psi. The declustering potential (DP) and collision energy (CE) for individual MRM transitions were determined with further DP and CE optimization. A specific set of MRM transitions was monitored for each period according to the metabolites eluted within this period.

### Transcriptome Sequencing and Transcript Quantification

Three biological replicates of TW (pool of five trees), OW (pool of five trees), and NW (pool of five trees) were used to carry out multiomic sequencing. Total RNA from the TW, OW, and NW of each sample was isolated using an RNA reagent kit (DP441; Tiangen Biotech, Beijing, China) following the manufacturer’s protocol. RNA quality was assessed on an Agilent 2100 bioanalyzer (Agilent Technologies, Palo Alto, CA, United States) and checked using RNase-free agarose gel electrophoresis. In total, nine cDNA libraries (three libraries each for TW, OW, and NW) were constructed for mRNA and small RNA high-throughput sequencing using an Illumina HiSeq^TM^ 4000 platform (Illumina, United States) by Gene *Denovo* Biotechnology Co. (Guangzhou, China). Each base in the reads was assigned a quality score (Q) with a Phred-like algorithm using SOAPnuke software to assess the quality of the RNA-sequencing (RNA-seq) data (Li et al., 2012).

High-quality clean reads were obtained by removing adaptor sequences. RSEM software was used to quantify transcripts (Li and Dewey, 2011). Fragments per kilobase of transcripts per million mapped reads (FPKM) were used to normalize transcript expression. The transcripts with a fold change ≥ 1.5, a *p*-value < 0.05, and an FPKM > 1 in a comparison were significant differentially expressed genes (DEGs).

### Protein Extraction and Labeling

Xylem samples of TW, OW, and NW were ground in liquid nitrogen into powder and transferred to 2 mL of lysis buffer [8 M urea, 2% SDS, 1 × Protease Inhibitor Cocktail (Roche Ltd., Basel, Switzerland)] and sonicated on ice for 30 min, and the homogenate was centrifuged for 30 min at 13,000 rpm and 4°C. The supernatant was transferred to a fresh tube. For each sample, proteins were precipitated with ice-cold acetone at −20°C overnight. The precipitates were washed with acetone three times and redissolved in 8 M urea by sonication on ice. Protein quality was examined by SDS-PAGE (Supplementary Figure 1).

Then, 100 mL of 100 mM triethylammonium bicarbonate (TEAB) and 5 μL of 200 mM Tris(2-carboxyethyl)phosphine (TCEP) were added per 100 μg of protein. The mixture was incubated at 55°C for 1 h. Then, 5 μL of 375 mM iodoacetamide was added and incubated for another 30 min. Ice-cold acetone was added to precipitate proteins, which were then redissolved in 100 μL TEAB. Proteins were digested with trypsin (Promega, Madison, WI, United States), and the peptide mixture was labeled with tandem mass tags (TMT). The peptide mixture was redissolved in 20 mM ammonium formate solution, pH 10.0, and then an Ultimate 3000 system (Thermo Fisher Scientific, Waltham, MA, United States) was used to fractionate the mixture by high-pH separation *via* a linear gradient. The column was re-equilibrated under initial conditions for 15 min. The column flow rate was maintained at 1 mL/min, and the column temperature was maintained at 30°C. See the literature (Xiao et al., 2020) for more details.

### Protein Identification and Quantification

Tandem mass spectra were extracted, charge state deconvoluted and deisotoped by Mascot Distiller version 2.6. Then, the mass spectrometry data were transformed into mascot generic file (MGF) files with Proteome Discovery 1.2 (Thermo Fisher Scientific, Pittsburgh, PA, United States) and analyzed using the Mascot search engine (Matrix Science, London, United Kingdom; version 2.3.2). The Mascot database was set up for protein identification using the *C. bungei* reference transcriptome assuming the digestion enzyme trypsin with one missed cleavage allowed. Mascot was searched with a fragment ion mass tolerance of 0.050 Da and a parent ion tolerance of 20.0 ppm. Carbamidomethyl of cysteine and iTRAQ8plex of lysine and the n-terminus were specified in Mascot as fixed modifications. Deamidation of asparagine and glutamine and oxidation of methionine and acetyl groups at the n-terminus were specified in Mascot as variable modifications.

Protein identifications were accepted if they could achieve an false discovery rate (FDR) <1.0% by the scaffold local FDR algorithm. Proteins were classified to satisfy the principles of parsimony if they contained similar peptides and could not be differentiated based on MS/MS analysis alone. In total, 17,548 unique peptides corresponding to 5,366 proteins were quantified. Proteins identified with unique spectra ≥2 in all the samples were used for quantification. The ratios of reporter ions reflect the relative abundances of peptides to ensure relative protein quantification. Medians were used to average and quantify the Mascot search results. Proteins with a fold change >1.2 or <0.83 in a comparison and with an unadjusted significance level *P* < 0.05 were significant differentially expressed proteins (DEPs).

### Prediction of Key Transcription Factors

The DEGs related to lignin monomer synthesis between TW and OW or between TW and NW were selected. TBtools (Chen et al., 2020) was used to extract 2,000-bp sequences before the CDSs of key genes as potential promoter sequences based on the *C. bungei* genome data. The promoter sequences of functional genes were imported into the online database PlantTFDB,^1^ and the potential transcription factors of target genes were predicted and regulated based on the Arabidopsis genome. The best match between Arabidopsis and *C. bungei* was found by basic local alignment search tool (BLAST). Then, we analyzed the differential expression of TFs between TW and OW and between TW and NW. The significantly differentially expressed TFs were candidate TFs.

### Statistical Analysis

SPSS 22.0 was used for variance analysis to test the significant differences in metabolite content and gene expression among different wood types. The Duncan method was used for multiple comparisons after variance analysis. GraphPad Prism 6.0 was used to draw scatter plots of gene expression and linear regression analysis. Pearson correlation analysis of the gene transcription and translation levels was carried out by SPSS 22.0, and *P* < 0.05 was the threshold for significance.

## Results

### The Anatomy of Tension Wood, Opposite Wood, and Normal Wood

**FIGURE 1:**
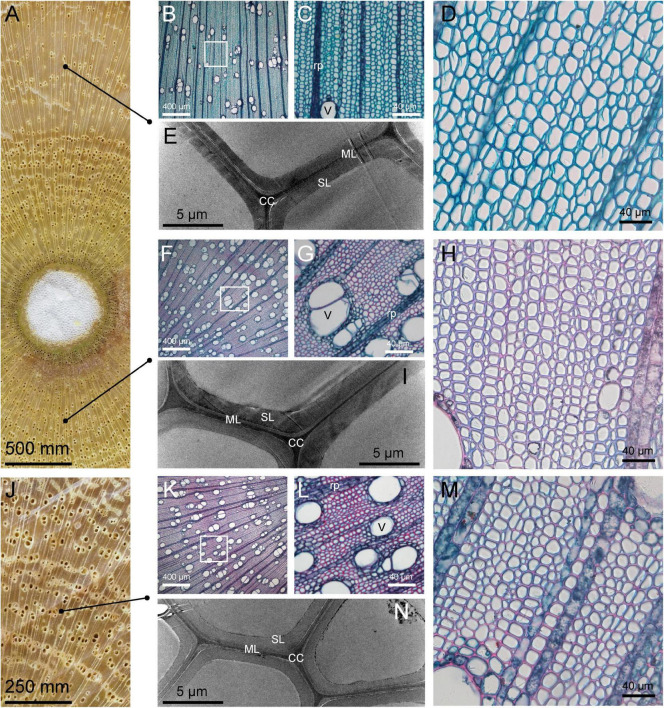
Microstructure and ultrastructure of tension wood (TW), opposite wood (OW), and normal wood (NW). The tissue sections were stained with safranine and fast green. **(A)** Transverse TW and OW. **(B–D)** Light microscopy cross sections of TW. **(E)** Transmission electron microscopy illustrating TW; **(F–H)** light microscopy cross sections of OW. **(I)** Transmission electron microscopy illustrating OW; **(J)** transverse NW. **(K–M)** Light microscopy cross sections of NW. **(N)** Transmission electron microscopy illustrating of NW. rp, ray parenchyma; SL, secondary wall layer; ML, middle lamella; CC, cell corner; V, Vessel.

Observation of the transverse section morphology of the *C. bungei* xylem showed that the differentiation of TW vessels was greatly affected. The number of vessels was significantly reduced, and their size was smaller than those in OW and NW (Figure 1). TW had a thinner cell wall than OW and NW according to the ultrastructure of the cell wall and the statistical results for cell wall thickness in our previous study (Xiao et al., 2020). Slice staining showed that TW could be dyed with fast green (Figures 1B,C), indicating lower lignification of TW. These results also suggested that the formation of the TW cell wall was inhibited and that its chemical composition might be greatly changed.

### Raman Spectroscopic Imaging and Microspectroscopy of Tension Wood, Opposite Wood, and Normal Wood

**FIGURE 2:**
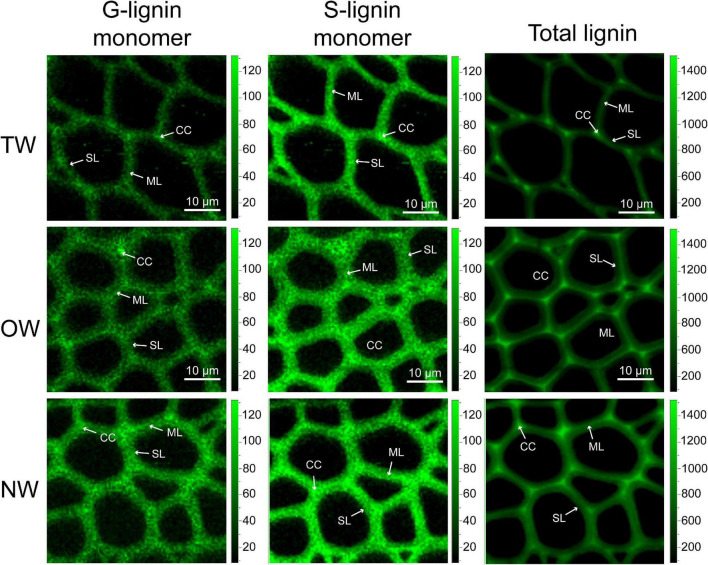
Confocal Raman spectral imaging of tension wood (TW), opposite wood (OW), and normal wood (NW). Confocal Raman images of G-lignin monomer at 1,275 wavenumber/cm^–1^, S-lignin monomer at 1,337 wavenumber/cm^–1^, and total lignin at 1,607 wavenumber/cm^–1^. SL, secondary wall layer; ML, middle lamella; CC, cell corner.

**FIGURE 3:**
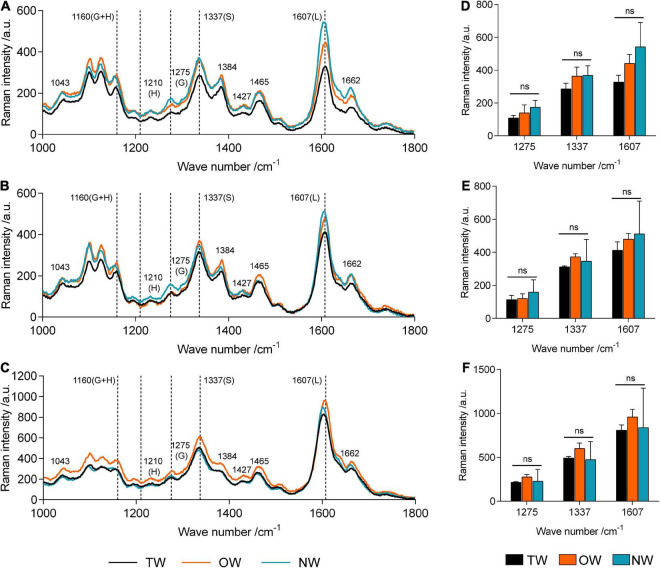
Averaged Raman spectra in the range of 1,000–1,800 cm^–1^ in different cell wall layers of tension wood (TW), opposite wood (OW), and normal wood (NW). **(A)** Averaged Raman spectra of the secondary wall layer, **(B)** averaged Raman spectra of the middle lamella, and **(C)** averaged Raman spectra of the cell corner. **(D–F)** Raman intensities at 1,275, 1,337, and 1,607 of TW, OW, and NW in the secondary wall layer, middle lamella, and cell corner, respectively. G, G lignin; H, H lignin; S, S lignin; L, total lignin; ns, not significant. Duncan’s method was used for multiple comparisons.

Confocal Raman microscopy was used to obtain more information about lignin in different cell wall regions of TW, OW, and NW. Confocal imaging showed that the total lignin signal intensity from OW and NW fibers was greater than that from TW. This indicated decreased lignin deposition in TW (Figure 2). Figure 3 shows that the Raman spectral intensities of the secondary layer (SL), middle layer (ML), and cell corner (CC) of TW were lower than those of OW and NW. According to previous literature (Table 1), characteristic Raman peaks at 1,210, 1,275, and 1,337 cm^–1^ reflect H, G, and S lignin, respectively. There was no obvious peak at 1,210 cm^–1^ from the fiber cell wall in this study. However, there was a weak peak at 1,275 cm^–1^ and a strong peak at 1,337 cm^–1^, which indicated a high relative content of S lignin but no H lignin in *C. bungei* wood.

**TABLE 1:** The Raman bands used for analysis and their assignment to lignins.

Wavenumber/cm^–1^	Band assignment	References
1,043	OC stretch, ring deformation, CH3 wagging	Gierlinger and Schwanninger, 2006
1,143–1,209	Lignin hydroxyl COH bend, lignin methoxy vibrations	Agarwal et al., 1997
1,272	Ring deformation, CO stretch	Wiley and Atalla, 1987; Saariaho et al., 2005
1,331	Aliphatic OH stretch	Wiley and Atalla, 1987; Agarwal et al., 1997; Edwards et al., 1997
1,378–1,390	Phenolic OH stretch, CH bend	Edwards et al., 1997
1,427	Lignin methoxy deformation, methyl bending, aromatic skeletal vibration	Saariaho et al., 2005
1,465	CH3 out-of-phase bend, CH2 scissoring	Edwards et al., 1997
1,600	Lignin aromatic skeletal vibrations	Edwards et al., 1997; Gierlinger and Schwanninger, 2006
1,656	Lignin CC stretch of coniferyl alcohol and sinapyl alcohol	Agarwal et al., 1997; Saariaho et al., 2005

The peak at 1,607 cm^–1^ reflects the presence of aromatic lignin skeletal vibrations. In the SL, ML, and CC, the Raman intensity of this peak from OW was approximately 37.27, 14.42, and 15.64%, respectively, higher than that from TW. The Raman intensity of this peak from NW was also higher than that from TW, but the standard deviation in these intensities was large. This result suggests that the difference in lignin deposition between TW and OW or NW is mainly reflected in the S layer. Similarly, the characteristic peak at 1,275 cm^–1^, which reflects G lignin in the ML layer, was not different between TW and OW, while in the S layer, there was a 28.31% higher value for OW than for TW. Although the intensity of the peak at 1,275 cm^–1^ in the ML was different between TW and NW, there was a slightly large error for NW; thus, we could not accurately assess the differences. The results above further confirmed that the lignin metabolism difference in TW fibers mainly occurred in the SL. The Raman intensities of each functional group in lignin molecules in the CC layer were generally higher than those in the other layers, indicating that the lignin deposition in TW, OW, and NW began in the CC layer, and these peaks for OW were all greater than those for TW. Raman confocal imaging also clearly showed the strongest Raman spectral signals at 1,250–1,290 cm^–1^, 1,320–1,338 cm^–1^, and 1,590–1,620 cm^–1^ in CC. This implied that lignin synthesis and deposition in TW were inhibited from the beginning (Figure 3C).

### LC-MS of Metabolites in the Lignin Monomer Biosynthesis Pathway in Tension Wood, Opposite Wood, and Normal Wood

**FIGURE 4:**
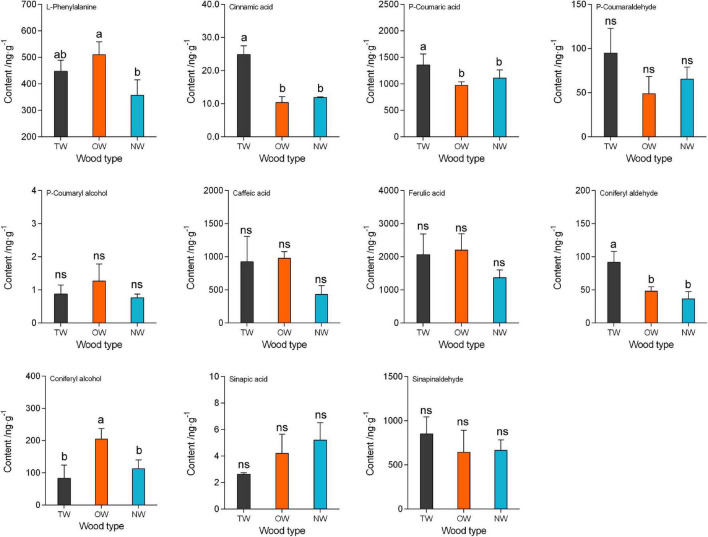
The contents of metabolites in lignin monomer biosynthesis in tension wood (TW), opposite wood (OW), and normal wood (NW). Duncan’s method was used for multiple comparisons. Different small letters indicate 5% significant differences. Different lowercase letters indicate a 5% significant difference. ns, not significant.

To reveal the change in lignin metabolism during TW formation in *C. bungei*, we used LC-MS to determine the contents of metabolites in the monolignol synthesis pathways in TW, OW, and NW (Figure 4). The results showed that the content of *p*-coumaryl alcohol was very low in these three types of wood tissues, but coniferyl alcohol and sinapyl aldehyde in TW, OW, and NW were more abundant than *p*-coumaryl alcohol. This suggested that H lignin was almost absent from the fiber cell wall of *C. bungei*, and S lignin was dominant, which was consistent with the Raman spectroscopy results. Cinnamic acid and *p*-coumaric acid in TW were significantly more abundant than those in OW and NW. Interestingly, we found that both *p*-coumaryl alcohol and coniferyl alcohol were less abundant in TW than in OW, and the content of coniferyl alcohol in TW was significantly lower (41% of that in OW) than that in OW. Unfortunately, this study did not determine the amount of sinapyl alcohol, but we inferred that its content in TW would be lower than that in OW. The main reason why the total lignin content of TW was significantly lower than that of OW was the reduced biosynthesis of cinnamyl alcohol in TW.

### KEGG Pathway Enrichment of Differentially Expressed Genes and Differentially Expressed Proteins

Kyoto encyclopedia of genes and genomes (KEGG) pathway enrichment results showed that DEGs were significantly enriched into the phenylpropanoid biosynthesis pathway. The enrichment *Q*-value of this pathway was 0.01 and <0.001 in TW vs. NW and TW vs. OW, respectively (Supplementary Figure 2A). On the other hand, DEPs in TW vs. OW were also significantly enriched to phenylpropanoid biosynthesis (*Q*-value = 0.014) (Supplementary Figure 2C). The rich factor represents the proportion of DEGs in a metabolic pathway to the total number of genes, and the larger the value is, the higher the enrichment degree is. The rich factor of phenylpropanoid biosynthesis of DEGs was relatively high in TW vs. NW (0.06) and TW vs. OW (0.07) (Supplementary Figure 2B). And the rich factor of phenylpropanoid biosynthesis of DEPs in TW vs. OW was 0.13, it was higher than that in TW vs. NW (0.06) (Supplementary Figure 2D). This indicated the phenylpropanoid biosynthesis was more important for the difference between TW and OW. All of the above results showed the change of phenylpropanoid biosynthesis would influence the TW information.

### Transcription and Translation Levels of Genes Involved in Lignin Monomer Biosynthesis in Tension Wood, Opposite Wood, and Normal Wood

**FIGURE 5:**
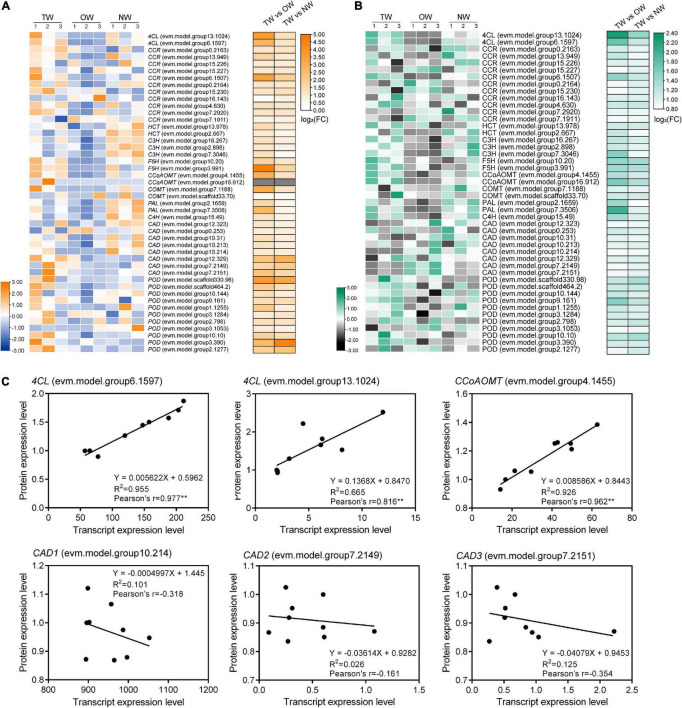
Transcription and translation levels of genes involved in lignin monomer biosynthesis in tension wood (TW), opposite wood (OW), and normal wood (NW). The numbers in panels **(A,B)** represent the biological replicates. **(A)** Transcription levels in TW, OW, and NW and differential expression of genes involved in the lignin metabolism pathway between groups, **(B)** translation levels in TW, OW, and NW and differential expression of genes involved in the lignin metabolism pathway between groups, **(C)** correlation analysis of the transcription and translation levels of candidate genes. Pearson’s r indicates the Pearson correlation coefficient. ** indicates 1% significance. Gene transcription levels and protein expression levels showed in heatmap were normalized.

The expression of genes and proteins involved in lignin biosynthesis was detected by transcriptome and proteome analyses. Detailed functional annotations of these genes can be found in Supplementary Table 1. Comparison of the expression levels of these genes or proteins between TW and OW and between TW and NW revealed that most of these genes had high levels of both transcription and translation in TW (Figure 5). The expression of *4CL* (evm.model.group13.1024), *PAL* (evm.model.group 7.3506), *CCoAOMT* (evm.model.group4.1455), *CCR* (evm.model.group6.1507), and *F5H* (evm.model.group3.991) significantly differed between TW and OW according to the transcriptome results (Figure 5A). Among them, 4CL and PAL had high levels of translation in TW according to the proteome (Figure 5B). Notably, most *CADs* in TW had higher transcription than those in OW and NW, but the translation of CADs in TW was lower than that in OW and NW. Correlation analysis of the gene expression levels showed that the transcription and translation levels of the two 4CLs and CCoAoMT genes were both significantly positively correlated (0.816–0.977). The transcription levels of *4CL* (evm.model.group6.1597) and *CCoAOMT* (evm.model.group4.1455) showed an obvious linear correlation with their translation levels (Figure 5C). In contrast, there was no obvious linear relationship between the transcription and translation levels of the three *CADs*, and the Pearson correlation of their transcription and translation levels was also weak.

**FIGURE 6:**
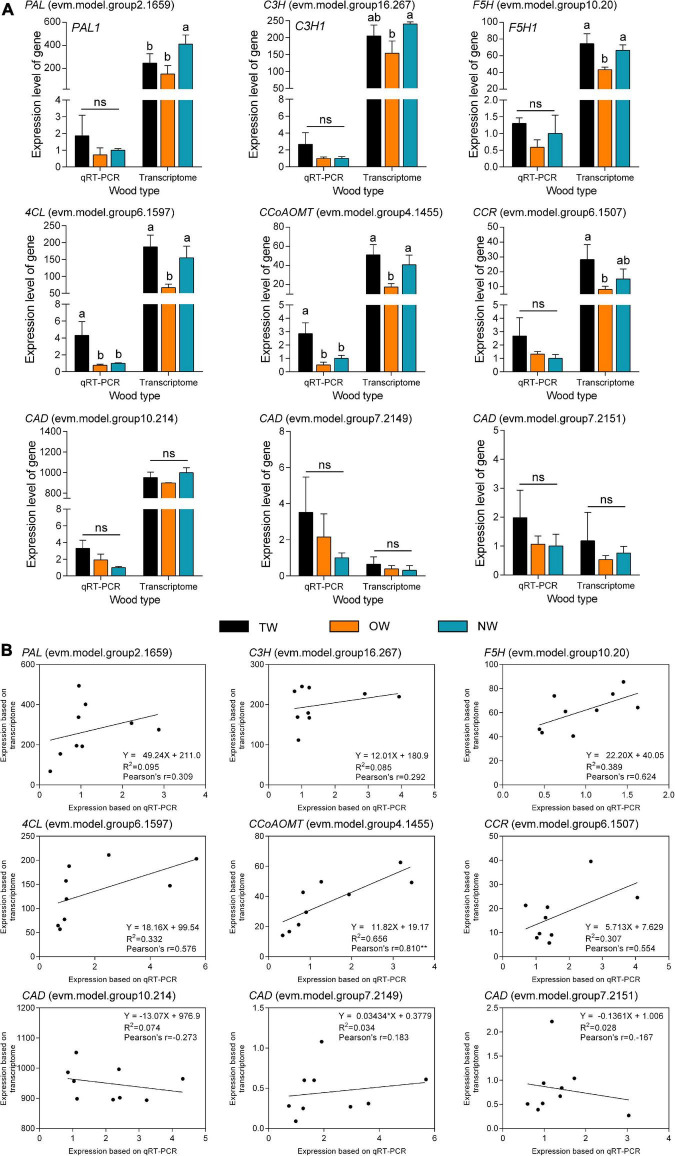
qRT-PCR validation of transcripts involved in lignin monomer biosynthesis in tension wood (TW), opposite wood (OW), and normal wood (NW). **(A)** qRT-PCR of candidate genes. **(B)** Correlation analysis of qRT-PCR and transcriptome results for candidate genes. Duncan’s method was used for multiple comparisons. Different lowercase letters indicate a 5% significant difference. ns, not significant. Pearson’s r indicates the Pearson correlation coefficient. ** indicates 1% significance. The expression of NW was as the control in qRT-PCR experiment.

**FIGURE 7:**
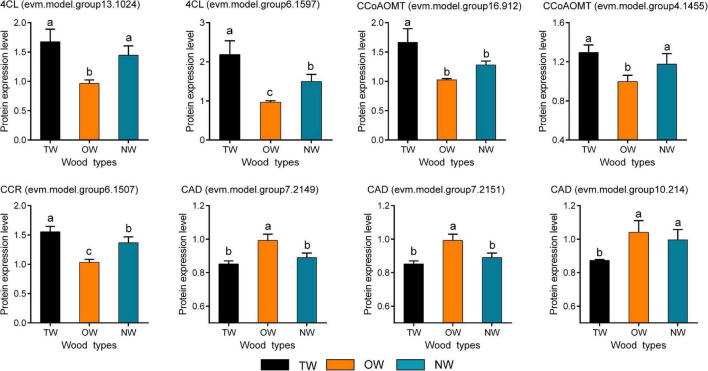
The expression of candidate proteins involved in lignin monomer biosynthesis in tension wood (TW), opposite wood (OW), and normal wood (NW). Duncan’s method was used for multiple comparisons. Different lowercase letters indicate a 5% significant difference. One of the biological repetitions of OW serves as a reference for calculating protein relative expression.

Quantitative Real-time polymerase chain reaction (qRT-PCR) was used to validate the results of RNA-seq (Figure 6A). The transcription level of *4CL* (evm.model.group6.1597) in TW was significantly higher than that in OW and NW. This result was consistent with the transcription level of *CCoAOMT* (evm.model.group4.1455). Correlation analysis showed that the results of the qRT-PCR of *CCoAOMT* were positively correlated (0.810) with its FPKM value from the transcriptome. On the other hand, the transcription levels of the three *CAD* genes were not significantly different among TW, OW and NW. However, their qRT-PCR results were weakly correlated with the transcriptome sequencing results (Figure 6B), which may have been due to the specificity of primers or the difference in RNA samples used in this experiment. In contrast, two *4CLs* (evm.model.group13.1024, evm.model.group6.1597) and one *CCR* (evm.model.group6.1507) showed significantly higher transcription and translation levels in TW than in OW and NW. The translation level of 4CL (evm.model.group6.1597) in TW was 1.50 times that in OW and 1.14 times that in NW. However, the translation level of the three CADs in TW was significantly lower than that in OW, and the expression level of CAD (evm.model.group10.214) in TW was significantly lower than those in OW and NW (Figure 7). The translation levels of the three CADs in OW were approximately 16.38–19.20% higher than those in TW (Figure 7 and Supplementary Table 2).

Proteins are better for explaining biological problems as the basis for their function in live organisms. Therefore, the significantly low level of CAD enzymes in TW was probably the main factor limiting monolignol biosynthesis, which eventually led to a decrease in lignin deposition. We further mapped the 17 DEGs whose transcription and translation levels were both significantly different in the lignin monomer metabolic pathways (Supplementary Figure 3). This figure visually shows the transcription and translation levels of genes involved in each chemical reaction in the lignin monomer synthesis pathway during TW formation, increasing the understanding of the mechanism of lignin monomer biosynthesis in *C. bungei*.

### Transcriptional Regulatory Network of Lignin Monomer Biosynthesis in Tension Wood

Transcription factors are some of the most important molecules that regulate gene expression. We used the PlantTFDB online database to predict the transcription factors that potentially regulate 17 candidate genes involved in lignin monomer synthesis. On the basis of the targeted relationship, we selected the differentially expressed transcription factors in TW and OW or in TW and NW as candidate regulatory genes (Table 2). In this study, 14 transcription factors were identified. Unfortunately, these 14 proteins were not identified in the proteome. Therefore, only their transcriptional levels are shown here. HD-ZIP (evm.model.group7.1157), LBD (evm.model.scaffold464.1), MYB (evm.model.group2.1745), MYB_related (evm.model.group2.388), and NAC (evm.model.group1.695) were significantly differentially expressed in TW and OW, and the remaining transcription factors were significantly differentially expressed in TW and NW.

**TABLE 2:** The expression and annotation of candidate transcription factor-regulated genes involved in lignin monomer biosynthesis.

Gene ID	Family	Transcriptional expression (mean ± SD)			Different expression (TW/OW)		Different expression (TW/NW)		Node degree	Best hit in *A. thaliana*	Description
		TW	OW	NW	log_2_(FC)	*P*-value	log_2_(FC)	*P*-value			
evm.model.group0.1192.1	ARR-B	3.08 ± 1.22	5.67 ± 1.5	6.79 ± 0.96	−0.88	/	−1.14	0.011	3	AT4G16110	Response regulator 2
evm.model.group1.1335	bHLH	3.91 ± 1.14	1.25 ± 0.8	0.22 ± 0.11	1.65	/	4.15	<0.001	11	AT3G59060	Phytochrome interacting factor 3-like 6
evm.model.group13.293	bHLH	7.04 ± 3.69	10.79 ± 2.96	15.41 ± 4.15	−0.62	/	−1.13	0.003	9	AT5G08130	bHLH family protein
evm.model.scaffold294.22	C2H2	17.27 ± 10.8	11.76 ± 9.87	5.91 ± 0.21	0.55	/	1.55	0.004	6	AT1G27730	Salt tolerance zinc finger
evm.model.group19.297	ERF	7.55 ± 6.76	9.21 ± 4.92	2.55 ± 0.52	−0.29	/	1.57	0.039	7	AT4G17490	Ethylene responsive element binding factor 6
evm.model.group7.1157	HD-ZIP	46.08 ± 7.01	21.36 ± 2.57	25.51 ± 3.61	1.11	<0.001	0.85	/	10	AT2G46680	Homeobox 7
evm.model.group3.142	HD-ZIP	26.04 ± 5.23	30.17 ± 7.81	53.29 ± 5.64	−0.21	/	−1.03	<0.001	6	AT4G40060	Homeobox protein 16
evm.model.scaffold464.1	LBD	12.41 ± 4.62	3.5 ± 1.76	10.82 ± 3.66	1.83	0.006	0.20	/	6	AT2G30340	LOB domain-containing protein 13
evm.model.group2.1745	MYB	1.92 ± 0.61	0.07 ± 0.07	0.85 ± 0.14	4.78	0.011	1.18	/	7	AT3G53200	MYB domain protein 27
evm.model.group2.388	MYB_related	2.92 ± 1.81	9.31 ± 5.16	0.77 ± 0.54	−1.67	0.042	1.92	/	5	AT5G56840	MYB_related family protein
evm.model.group1.695	NAC	16.75 ± 3.08	6.57 ± 0.9	13.27 ± 3.06	1.35	<0.001	0.34	/	7	AT4G28500	NAC domain containing protein 73
evm.model.group1.1356	NAC	4.55 ± 4.33	4.71 ± 2.08	13.53 ± 7	−0.05	/	−1.57	0.021	8	AT2G43000	NAC domain containing protein 42
evm.model.group1.1859	NAC	5.44 ± 1.55	5.61 ± 1.48	2.26 ± 0.59	−0.04	/	1.27	0.045	4	AT3G15510	NAC domain containing protein 2
evm.model.group0.1498	WRKY	21.34 ± 2.63	13.28 ± 1.89	6.71 ± 1.58	0.68	/	1.67	<0.001	4	AT1G69310	WRKY DNA-binding protein 57

*FC, Fold change; TW, Tension wood; OW, Opposite wood; NW, Normal wood.*

**FIGURE 8:**
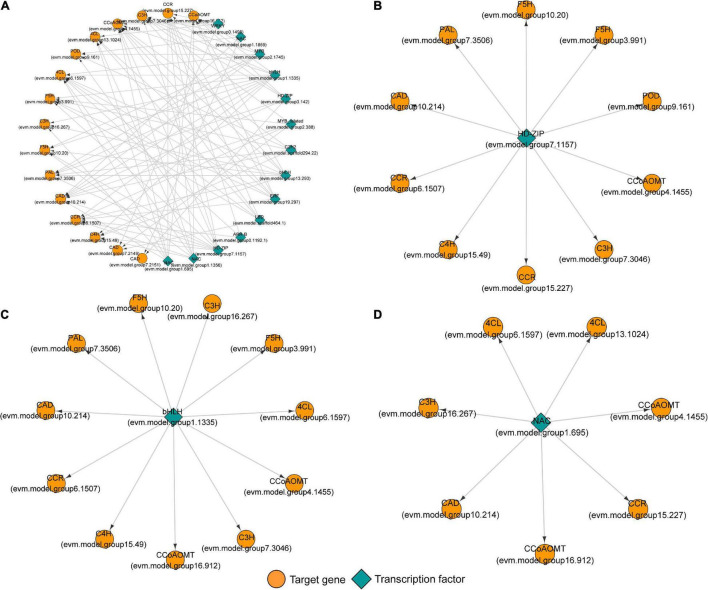
The potential regulatory network between transcription factors and genes involved in lignin monomer biosynthesis in tension wood (TW) formation. **(A)** The regulatory network between candidate transcription factors and 17 key genes involved in lignin monomer biosynthesis. **(B)** The regulatory network of key HD-ZIP transcription factors and target genes. **(C)** The regulatory network of key bHLH transcription factors and target genes. **(D)** The regulatory network of key NAC transcription factors and target genes.

Figure 8A shows the regulatory relationship between the candidate transcription factor and 17 target genes. Each transcription factor has multiple target genes with a node degree between 3 and 11 (Table 2). The node degree could represent the importance of transcription factors in the metabolic pathway. The node degrees of bHLH (evm.model.group1.1335) and HD-ZIP (evm.model.group7.1157) were 11 and 10, ranking first and second, respectively. Thus, they are considered important transcription factors. They all had targeting relationships with the *CCoAOMT*, *F5H*, *CCR*, and *CAD* genes (Figures 8B,C). Second, we found that several genes that had a potential targeting relationship with NAC (evm.model.group1.695), including *4CL*, *CCoAOMT*, and *CCR*, were more directly related to cinnamaldehyde biosynthesis. Therefore, we believe that NAC is also an important regulatory factor affecting lignin monomer synthesis (Figure 8D). The matching sequence information of the three key transcription factors and target genes is shown in Supplementary Table 3.

**FIGURE 9:**
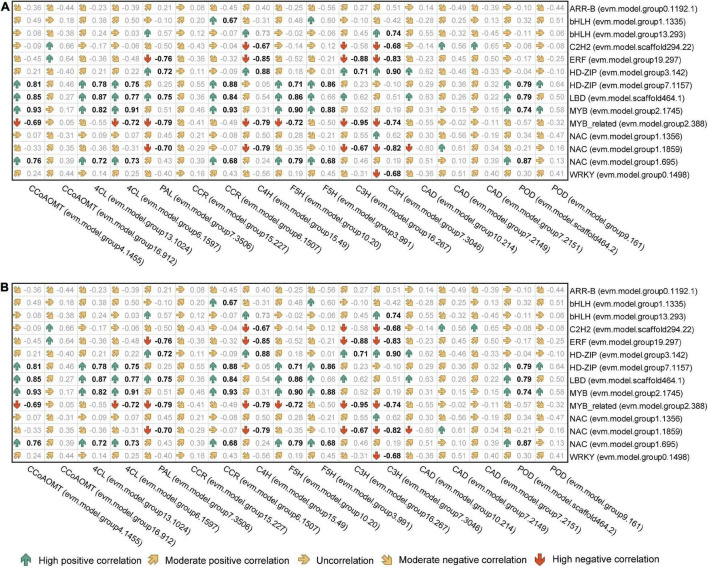
Correlation analysis of the expression levels between candidate transcription factors and genes involved in lignin monomer biosynthesis. **(A)** The correlation coefficient between the transcription level of transcription factors and functional genes. **(B)** The correlation coefficient between the transcription level of transcription factors and the translation level of functional genes. Bold black indicates a *P* < 0.05 significance level.

The correlation analysis of gene expression (Figure 9) showed that although bHLHs (evm.model.group1.1335) had a high node degree, the correlation between their expression level and target gene transcription and translation levels was weak. Therefore, bHLHs were not considered to have a critical regulatory role in the synthesis of lignin monomers. In contrast, the transcription level of *HD-ZIP* (evm.model.group7.1157) was significantly positively correlated with the transcription and translation levels of *CCoAOMT* (evm.model.group4.1455), *CCR* (evm.model.group6.1507) and *F5H* (evm.model.group3.991), and the correlation coefficient for the former two groups was >0.8. Similarly, *NAC* (evm.model.group1.695) was significantly positively correlated with the transcription and translation levels of *CCoAOMT* (evm.model.group4.1455) and two *4CLs* (correlation coefficient > 0.7) (Figure 9). *HD-ZIP* (evm.model.group7.1157) had a higher transcription level in TW (FPKM was 46.08), which was approximately twice that of OW and NW. It is speculated that this transcription factor is the most important transcription factor regulating lignin metabolism in *C. bungei* TW.

### Regulation Pattern Mediated by Transcription Factors in Lignin Biosynthesis in Tension Wood

**FIGURE 10:**
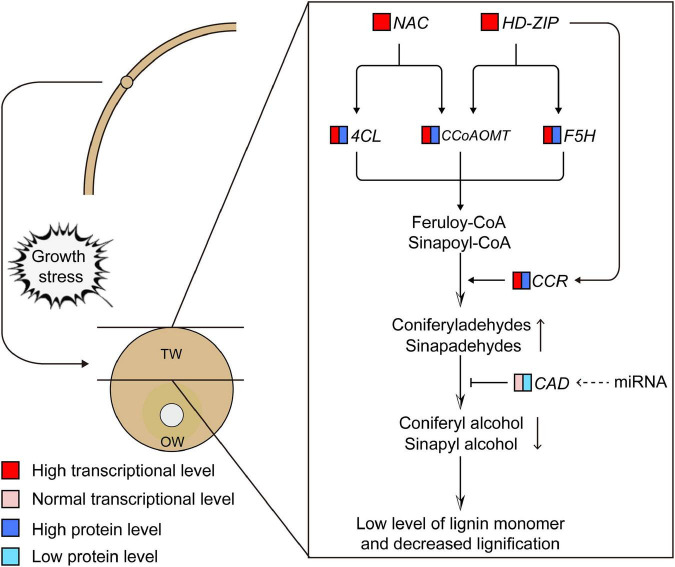
Transcriptional regulation model of lignin monomer biosynthesis during tension wood (TW) formation in *C. bungei*. The dotted line represents the uncertainty.

We preliminarily proposed the transcriptional regulation mechanism of lignin monomer synthesis of *C. bungei* TW according to the contents of metabolites in the lignin synthesis pathway, the transcription and translation of key genes and the potential regulatory relationship between candidate transcription factors and genes (Figure 10). When the stems of trees grew under stress, the expression levels of the NAC (evm.model.group1.695) and HD-ZIP (evm.model.group7.1157) transcription factors were significantly increased in the stress area. They could bind *4CL*, *CCoAOMT* or *F5H* genes and activate their expression at the transcription level, which may promote the production of feruloyl-CoA and sinapoyl-CoA. Then, HD-ZIP further promoted the expression of the *CCR* gene and enhanced the conversion of coenzyme A to cinnamaldehyde, significantly increasing the content of coniferyl aldehyde in TW. However, we speculated that the significantly lower protein content of the CAD gene in TW may inhibit the conversion of coniferyl aldehyde and sinapaldehydes into coniferyl alcohol and sinapyl alcohol, resulting in a significant decrease in lignin monomer and total lignin in TW. The normal transcription level of CAD and the significantly lower translation level in TW suggested that CAD may be regulated at the post-transcriptional level and affect the translation of CAD. However, the specific regulatory mechanism remains to be further explored.

## Discussion

### Differences in Lignin Metabolism Among Tension Wood, Opposite Wood, and Normal Wood of *Catalpa bungei*

Tension wood is wood with unique anatomical and physical characteristics. Its properties have created a starting point for the study of wood formation mechanisms. Lignin is one of the main chemical components in wood. Changes in its content and structure will affect the quality and utilization of wood. Methods of directionally changing lignin are always popular research topics. In this study, the characteristics of lignin metabolism were evaluated by various methods. Histochemical staining showed that the lignin content of TW was significantly lower than that of OW, which agreed with previous studies (Jin and Kwon, 2009; Brereton et al., 2011; Mizrachi et al., 2015). The presence of a gelatinous layer (G layer) with no or an extremely low lignin content is considered the result of decreased lignification of TW (Pilate et al., 2004). Our previous work showed that the TW of *C. bungei* does not have a G-layer (Xiao et al., 2020); therefore, there may be a special regulation pattern for lignin metabolism during TW formation in *C. bungei*.

For a more comprehensive analysis of the changes in lignin metabolism during TW formation, LC-MS and confocal micro-Raman spectroscopy were used to further analyze the metabolic differences among TW, OW, and NW in the lignin monomer biosynthesis of *C. bungei*. Confocal micro-Raman spectrochemical imaging can reflect the differences in chemical components between samples more accurately without any chemical treatment and can also directly show the spatial distribution of lignin at the subcellular level (Gierlinger and Schwanninger, 2006). Analysis with this technique revealed an obvious difference in lignin metabolism between TW and OW or NW. In TW, not only was the total lignin content lower than that in OW and NW, but the contents of S lignin and G lignin were also lower than those in OW and NW. The Raman images of TW in poplar (Gierlinger and Schwanninger, 2006), maple, beech, and oak (Lehringer et al., 2008) were similar to our images. Notably, our results showed that TW had a significantly higher level of coniferyl aldehyde but a significantly lower level of coniferyl alcohol than OW and NW. Coniferyl alcohol, as one of the precursors of lignin monomers, had a great influence on the total lignin content. We speculate that the enzymes that catalyze this chemical process might have led to this result. In addition, higher contents of p-coumaraldehyde and sinapyl aldehyde and a lower content of *p*-coumaryl alcohol in TW than in OW and NW were also observed, although the difference did not reach the level of statistical significance. The conversion of cinnamaldehyde to cinnamyl alcohol is the main inhibitor of TW lignin biosynthesis. Unfortunately, sinapyl alcohol was not measured in this study, which is important evidence for our hypothesis.

### Gene Expression Involved in Lignin Biosynthesis in the Tension Wood of *Catalpa bungei*

To test and verify our hypothesis, transcriptome and proteome sequencing of TW, OW, and NW were carried out. The results showed that most of the DEGs and proteins in the phenylpropanoid biosynthesis pathway between TW and OW were highly expressed in TW. The transcriptome of TW in *Betula luminifera* was similar to our transcriptome (Cai et al., 2018). However, the results were opposite for *Populus*, *Eucalyptus*, and *Betula platyphylla*, in which genes or proteins involved in lignin monomer synthesis were mostly downregulated during TW formation (Andersson-Gunneras et al., 2006; Chen et al., 2015; Mizrachi et al., 2015; Bygdell et al., 2017). This suggests that the expression of genes involved in lignin metabolism is highly dynamic during TW formation. In particular, we found that *4CL* (evm.model.group6.1597) and *CCoAOMT* (evm.model.group4.1455) had high transcription and translation levels in TW, and their transcription and translation levels were significantly positively correlated. Thus, we believe they might be the key to the change in TW lignin biosynthesis in *C. bungei*. Their high expression could lead to high contents of feruloyl-CoA and sinapoyl-CoA in TW. On the other hand, the high transcription and translation of *CCR* (evm.model.group6.1507) may be the reason for the high coniferyl aldehyde content in TW. However, in this study, we found that the content of lignin was lower in TW than in OW and NW, contrary to the increase in the expression of most genes involved in lignin monomer biosynthesis in TW. Interestingly, we found that the translation levels of the three CADs were significantly decreased in TW compared with OW, even though their transcription levels were not different between TW and OW.

Compared with mRNA, protein, as the final implementer of biological functions, plays a direct role in determining life processes. CAD is the key enzyme leading to the synthesis of coniferyl alcohol and sinapyl alcohol and catalyzes the final step of lignin monomer biosynthesis (Mansell et al., 1974). Therefore, the change in CAD translation levels may be a more important factor in the trait development of TW. Eudes et al. (2006) found that *AtCAD1* partially complemented the *A. thaliana CAD* mutant and resulted in the restoration of lignin units. Baucher et al. (1996) showed that the total lignin content was not changed in transgenic poplar trees with antisense inhibition of *CAD* gene expression compared with normal poplar, but the cinnamaldehyde level was significantly increased in the transgenic plants. Furthermore, an increase in cinnamaldehyde and an unchanging lignin content were observed in transgenic tobacco with inhibited expression of *CAD* (Halpin et al., 1994). Natural CAD mutants of pine showed not only high cinnamaldehyde levels but also a 9% decreased lignin content (Mackay et al., 1997). All these studies indicated that CAD plays an important role in lignin biosynthesis. According to our proteomic and LC-MS results, we believe that the low expression of CAD protein in TW resulted in inhibition of lignin monomer biosynthesis and a lower content of lignin in TW. Similarly, Mauriat et al. (2015) found that the CAD enzyme was less abundant in the TW than in OW of poplar. However, more information is needed to determine the difference between the transcription level and translation level of *CAD* genes in TW.

### Molecular Regulation of Lignin Biosynthesis in the Tension Wood of *Catalpa bungei*

Many transcription factors, such as MYBs and NACs, have been proven to regulate lignin biosynthesis, and this regulatory hierarchy is similar to that of cellulose (Zhou et al., 2009; Zhao and Dixon, 2011). In our study, we also found that NAC (evm.model.group1.695) could regulate the expression of *4CLs* and *CCoAOMTs* to influence lignin monomer biosynthesis. Yang et al. (2019) found that overexpressed WOOD-ASSOCIATED NAC DOMAIN protein 3 (PdWND3A) in *Populus deltoides* can increase the lignin content and syringyl and guaiacyl lignin (S/G) ratio of transgenic plants, while the expression of *F5H* was increased. This result suggested that NAC may regulate the expression of the *F5H* gene to change lignin monomer synthesis in poplar. A recent study showed that the Eucalyptus NAC transcription factor can bind to the promoter of the *CCoAOMT* gene to activate its expression, and overexpression of this gene in *A. thaliana* will greatly promote xylem development and lignin synthesis (Sun et al., 2021). With the development of molecular biology techniques, more knowledge about the lignin synthesis mechanism, beyond the NAC-MYB transcriptional cascade, has been revealed (Zhao, 2016). In poplar, HD-ZIP transcription factors are highly expressed in the developing xylem (Schrader et al., 2004). Moreover, HD-ZIP transcription factors have been identified as important determinants of vascular bundle differentiation in *A. thaliana* (Zhong and Ye, 2004). With further research, Roc8 (one HD-ZIP TF) was found to be able to target the expression of LAC, a lignin synthesis gene, in rice, while knocking out *OsRoc8* led to a significant decrease in the lignin content (Sun et al., 2020). These results supported our finding that there was a potential targeting relationship between HD-ZIP (evm.model.group7.1157) and several genes involved in lignin monomer synthesis, and the expression levels were significantly positively correlated with the target gene abundance. This implies that HD-ZIP plays a critical role in the wood formation of *C. bungei*. Interestingly, the high transcription level and low translation level of CAD in this study also suggest the existence of post-transcriptional regulation. And we guess maybe there were some miRNAs involved the regulation process, cause miRNAs were important to post-transcriptional regulation of genes. On the other hand, CAD is a key enzyme in the last step of the biosynthesis of lignin monomers, the low protein expression level of CAD is likely an important factor affecting the synthesis of lignin monomers when most lignin synthesis genes are highly expressed. Therefore, the hypothesis that miRNAs regulate CAD protein expression in translation inhibition and influence TW lignin metabolism is indeed worth exploring. Previous studies have shown that miRNAs are involved in the regulation of lignin metabolism; for instance, miR397 negatively regulates the LAC gene to change the lignin content of poplar (Lu et al., 2013). miR6433 influences the synthesis of S lignin by binding with the F5H gene (Fan et al., 2020). Overexpression of miR156 can reduce the S/G ratio in poplar (Rubinelli et al., 2013). All of the above results suggest a new level of complexity in lignin biosynthesis regulation.

## Conclusion

In this study, we analyzed the differences in lignin biosynthesis in *C. bungei* at the transcription, translation, metabolism, and histochemistry levels between TW and OW or NW. We found that NAC and HD-ZIP transcription factors may be the key transcription factors regulating the biosynthesis of lignin monomers in *C. bungei* TW by analyzing the matching relationship between the transcription factors and the promoter sequences of key genes and the correlation of their transcription and translation levels. The significantly increased expression of NAC and HD-ZIP in TW may activate the expression of the *4CL*, *CCoAOMT*, *F5H*, and *CCR* genes and enhance the production of coniferyl aldehyde in TW. In contrast, the significantly decreased translation of CAD genes in TW may be the reason for the significantly decreased content of coniferyl alcohol, which ultimately limits lignin monomer biosynthesis in TW. However, the regulatory mode that changes the translation level of the CAD gene in TW needs further research.

## Data Availability Statement

The RNA-seq datasets are available in Sequence Read Archive (SRA) in NCBI with the accession number PRJNA559964.

## Author Contributions

YX performed the experiments, processed, analyzed, and interpreted the data, and wrote the manuscript with contributions from the other authors. JL, FY, and TZ assisted with the interpretation of the data and the writing of the manuscript. WM, KZ, and HY prepared the plant material and assisted in the data analysis. NL and TZ carried out the manuscript revision. JW conceived the original research plan and assisted with the design and interpretation of the data. All authors contributed to the article and approved the submitted version.

## Conflict of Interest

The authors declare that the research was conducted in the absence of any commercial or financial relationships that could be construed as a potential conflict of interest.

## Publisher’s Note

All claims expressed in this article are solely those of the authors and do not necessarily represent those of their affiliated organizations, or those of the publisher, the editors and the reviewers. Any product that may be evaluated in this article, or claim that may be made by its manufacturer, is not guaranteed or endorsed by the publisher.

## Footnotes

1
http://planttfdb.gao-lab.org/

## Supplementary Material

Supplementary Figure 1 Protein SDS-PAGE.

Supplementary Figure 2 The KEGG pathway enrichment of differentially expressed genes (DEGs) and differentially expressed proteins (DEPs). Gray indicates missing values.

Supplementary Figure 3 Differentially expressed genes (DEGs) and Differentially expressed proteins (DEPs) involved in the lignin monomer synthesis pathways in tension wood (TW) vs. opposite wood (OW) and TW vs. normal wood (NW).
