# Supplementary figures and images for: Transcriptomic, Proteomic, and Metabolic Profiles of Catalpa bungei Tension Wood Reveal New Insight Into Lignin Biosynthesis Involving Transcription Factor Regulation
Source: Front Plant Sci. 2021 Nov 15;12:704262. doi: 10.3389/fpls.2021.704262 (PMC8634757; doi:10.3389/fpls.2021.704262)

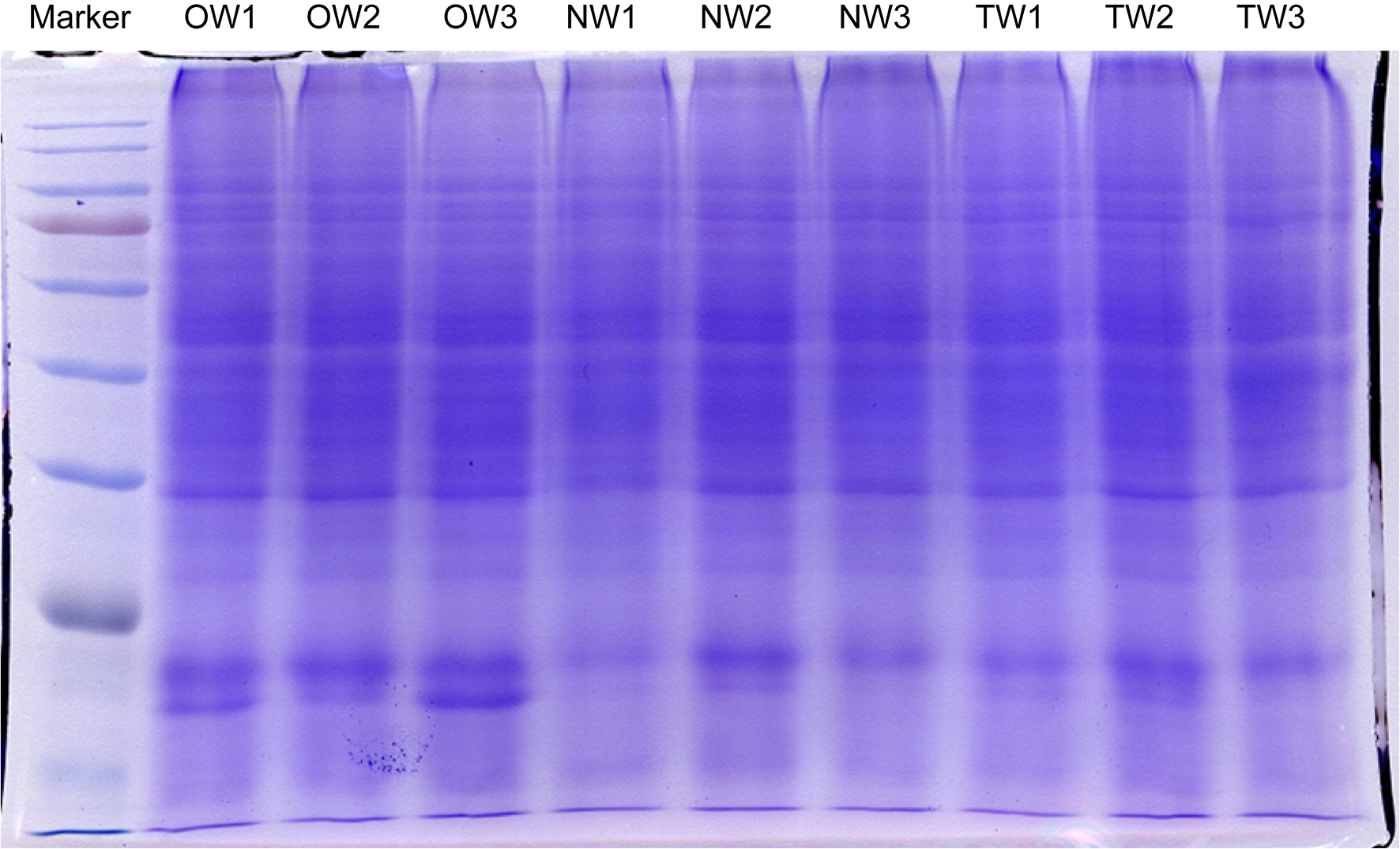

Supplement: Supplementary Figure 1 — Protein SDS-PAGE. [file Image_1.TIF]

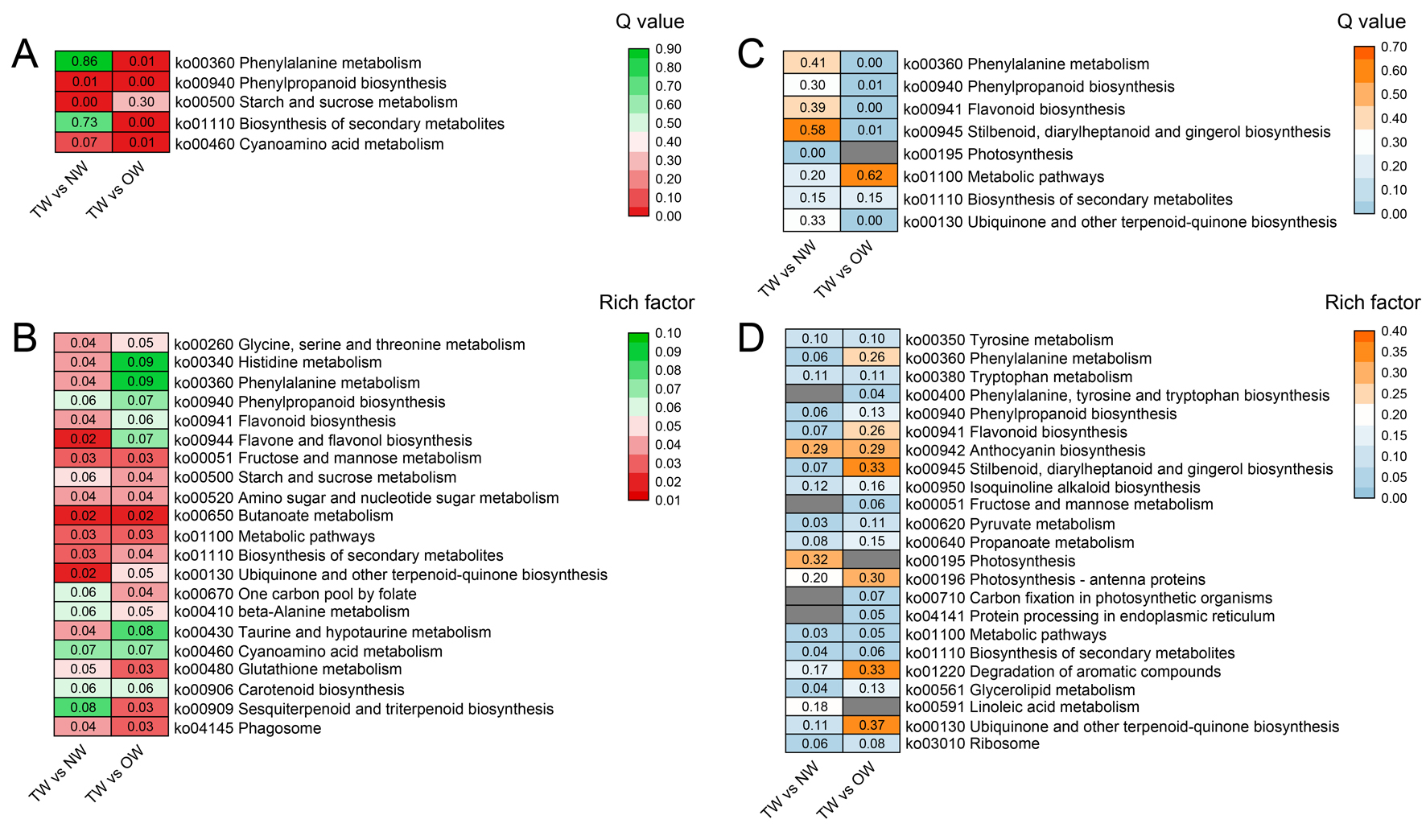

Supplement: Supplementary Figure 2 — The KEGG pathway enrichment of differentially expressed genes (DEGs) and differentially expressed proteins (DEPs). Gray indicates missing values. [file Image_2.JPEG]

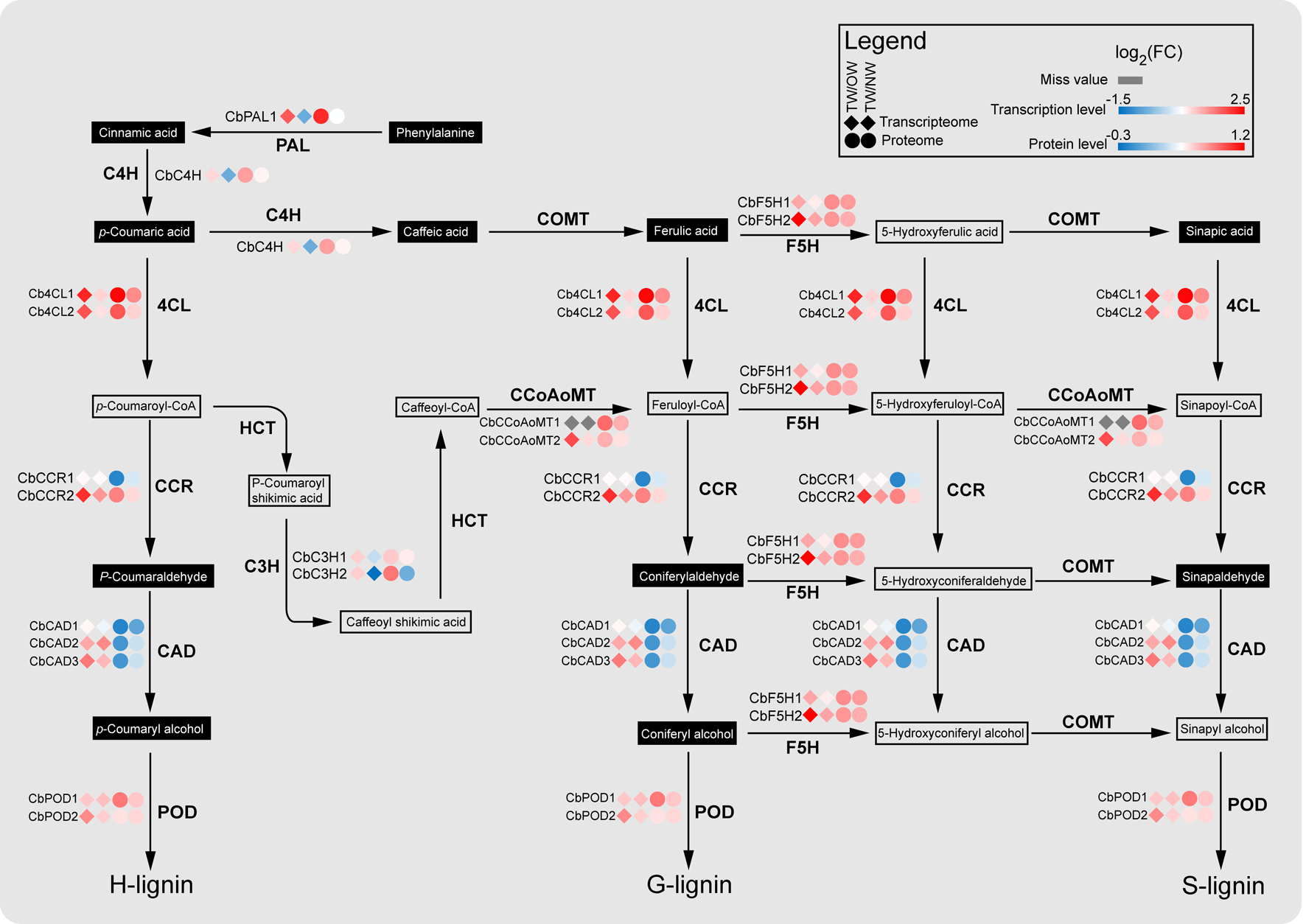

Supplement: Supplementary Figure 3 — Differentially expressed genes (DEGs) and Differentially expressed proteins (DEPs) involved in the lignin monomer synthesis pathways in tension wood (TW) vs. opposite wood (OW) and TW vs. normal wood (NW). [file Image_3.TIF]
